# Primary Mature Cystic Teratoma Compressing the Prostate in a 28-Year-Old Male: A Case Report and Literature Review

**DOI:** 10.1155/2019/8970172

**Published:** 2019-02-19

**Authors:** Khalil Chalhoub, Rawad Abou Zahr, Elias Mansour, Mona Aoun, Michel Jabbour

**Affiliations:** ^1^University of Balamand, Saint George Hospital University Medical Center, Department of Urology, Beirut 1100 2807, Lebanon; ^2^University of Balamand, Faculty of Medicine and Medical Sciences, Beirut 1100 2807, Lebanon; ^3^University of Balamand, Saint George Hospital University Medical Center, Department of Pathology, Beirut 1100 2807, Lebanon

## Abstract

Primary mature retroperitoneal teratomas are rare tumors most commonly occurring in adult females. These tumors are usually asymptomatic since they have no attachments to specific organs. We present a rare case of a 28-year-old male with 2-month history of lower urinary tract symptoms, who was found to have a primary mature cystic teratoma abutting the prostate.

## 1. Introduction

Dermoid cysts or mature teratomas are neoplastic structures composed of tissues from at least two of the three germ layers (endoderm, ectoderm, and mesoderm) [1, 2]. They are most commonly located in the gonads [3]. Primary retroperitoneal neoplasms comprise only 0.1%–0.3% of all the tumors, of which the most common are metastases from gonads, and 1%–11% of those are teratomas [4, 5]. Characteristically, mature teratomas are unilocular and, in addition to cutaneous components, may contain other tissues, including hair and teeth, glandular elements, adipose tissue, cartilage, and bone as well as sebaceous material [6]. Primary retroperitoneal teratomas (RT) characteristically have no attachment to other organs such as kidneys, adrenal glands, and pancreas, with the majority being asymptomatic or discovered incidentally on routine investigations [7, 8]. Surgical excision is the diagnostic modality of choice and the mainstay of treatment [9, 10]. Prognosis is excellent after complete surgical excision with an overall five-year survival rate of nearly 100% [11]. In the literature, few cases were reported, mostly occurring in infants or young females. The most common site is sacrococcygeal area in infants and left suprarenal region in adults [12, 13]. One case of mature teratoma involving the prostate was reported in a 50-year-old male with micturition problem. The aim of this article is to present a rare case of a 28-year-old male patient with psoriasis who sought medical care after 2-month history of urinary symptoms and increased bowel movements.

## 2. Case Presentation

A 28-year-old male patient with psoriasis presented to our facility with two-month history of lower urinary symptoms and increased bowel movements. Physical examination and laboratory studies were unremarkable. Suprapubic ultrasonography done outside our hospital showed an enlarged prostate measuring 72mm x 76mm x 67mm and 194 cm^3^ in volume. Pelvic Magnetic Resonance Imaging (MRI) showed a large mass confined to the pelvis measuring 7.2 cm in the largest diameter with predominance of cystic component without evidence of fatty content, calcification, fluid-fluid level, or suspicious enhancing nodular soft tissue thickening.

**Figure 1:**
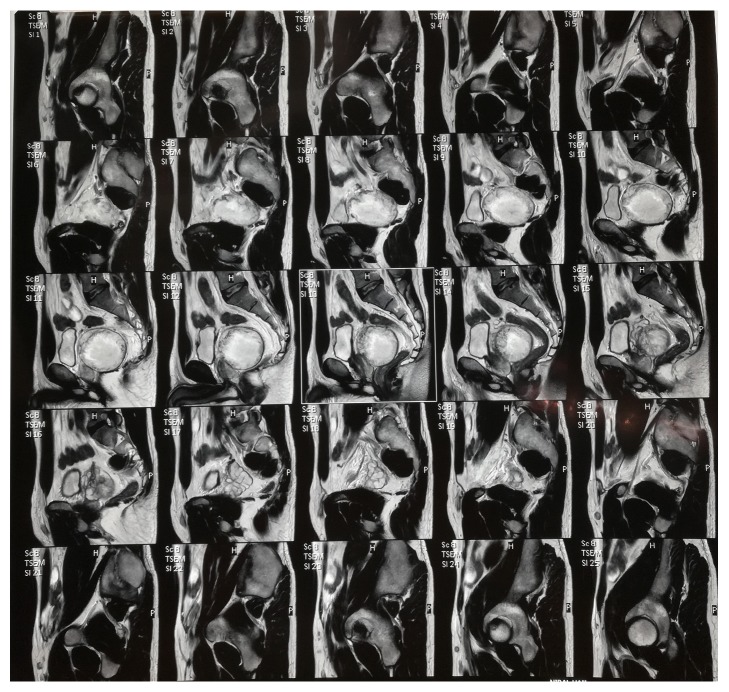
Pelvic MRI showing a large mass confined to the pelvis measuring 7.2 cm in the largest diameter with predominance of cystic component without evidence of fatty content, calcification, fluid-fluid level, or suspicious enhancing nodular soft tissue thickening.

The mass was seen in the perirectal space, displacing and exerting mass effect on the seminal vesicles, prostate and abutting the bladder without clear connection with the digestive system (Figure 1).

**Figure 2:**
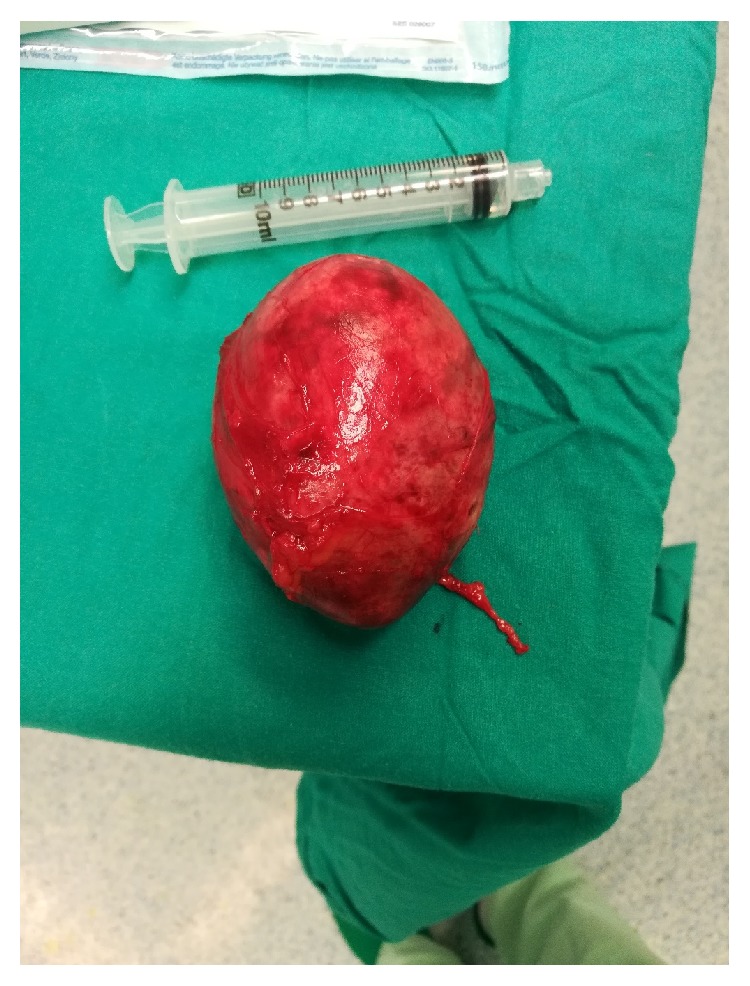
The excised mass had well-circumscribed smooth borders and rubbery consistency. It is covered by a thin and focally congested membrane. It weighs 157g and measures 7.5cm x 5.5cm x 5.5cm. Cut surface shows unilocular cystic mass filled with beige brown soft material and hair shafts.

Patient underwent a surgical resection through a suprapubic midline incision; the mass was approached from the left side after liberating and reflecting the bladder medially. Macroscopically, the mass weighs 157g and measures 7.5 x 5 x 5.5cm. It is covered by a thin membrane and is focally congested (Figure 2). Cut surface shows a unilocular cystic mass filled with beige brown soft material and hair shafts.

**Figure 3:**
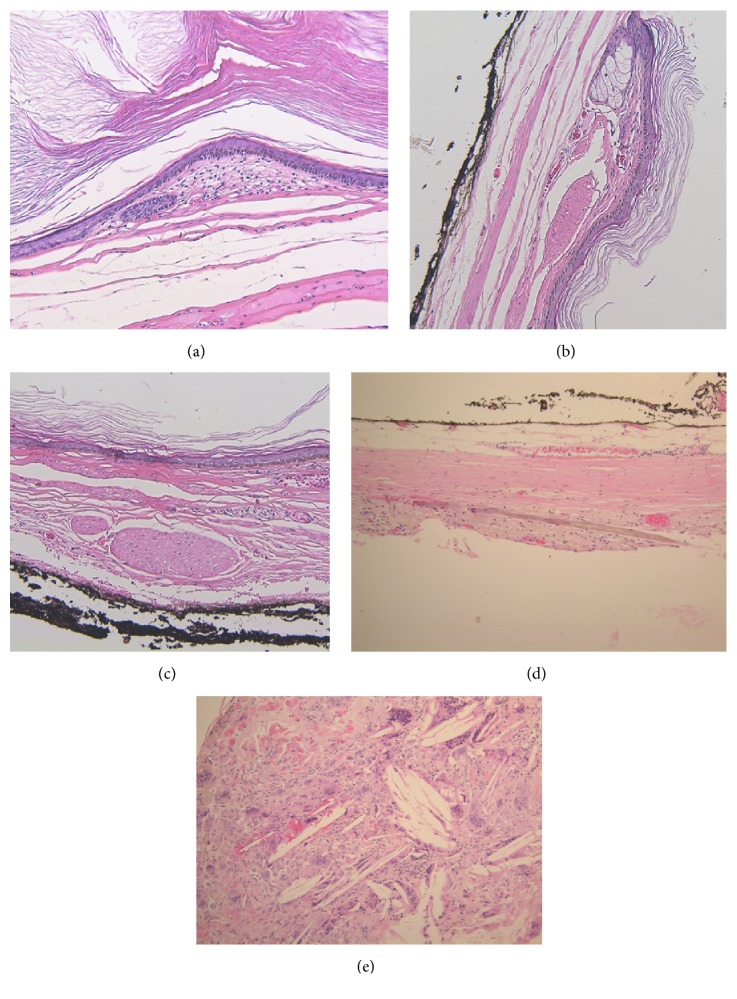
(a) & (b) Cystic cavity lined by orthokeratinized stratified squamous epithelium, with straight epithelial-connective tissue interface. Copious fibrillary keratin fills the cyst. (a) The base of a hair follicle is identified. (b) A sebaceous gland with adjacent arrector pili muscle fiber. (c) High power view of arrector pili muscle fiber. (d) A hair shaft found in the cystic wall. (e) Inflammatory reaction to keratin with multinucleated giant cells and foamy macrophages is seen. Absence of cartilage and bone in all figures.

Microscopically, the excised cystic mass is covered with a thin fibrotic wall and lined by mature squamous epithelium with few skin adnexae and hair shafts filled with fibrillary keratin. Pathology was consistent with mature cystic teratoma (Figure 3). Patient was discharged on the second postoperative day after an uneventful stay. Patient's urinary symptoms were relieved; MRI done at one year postoperatively showed no recurrence of tumor.

## 3. Discussion

Primary retroperitoneal teratomas constitute about 1-11% of all primary retroperitoneal tumors [14, 15]. The majority are diagnosed within the first year of life and 10-20% after 30 years of age. Adult retroperitoneal dermoid cysts commonly affect females between 15 and 40 years of age [16]. Embryologically, they originate from uncontrolled proliferation of pluripotent cells: germ cells and embryonal cells. Depending on the type of pluripotent cell, the presentation and location of teratoma may vary. Teratomas of germ cells are found in gonads (testes and ovaries). In contrast, teratomas of embryonic cell sources are always congenital and are usually found in extragonadal locations (intracranial, retroperitoneal, etc.) [17, 18]. Many theories account for the origin of the primary retroperitoneal teratomas; the most widely accepted one is that they are remnants of the Wolffian and Müllerian ducts or that they arise from pronephric or mesonephric tubules as it correlates with their midline and paramedian location [16].

Teratomas may be classified according to their location, content, epithelial lining, and degree of maturation [19]. Depending on the location, they can be gonadal or extragonadal with the former being more common. However, based on the content, teratomas can be solid, cystic, or mixed; furthermore, according to their epithelial lining, they can be divided into epidermoid, dermoid, and teratoid teratomas. Epidermoid and dermoid teratomas are lined with squamous epithelium but the latter contain dermoid elements (i.e., hair; sebaceous glands) while the teratoid type is lined by respiratory columnar epithelium and contains sebum. Besides, according to their degree of maturation, there are two types: mature and immature. Mature teratomas also called dermoid cysts are in general benign and are more common in females. The immature ones are poorly differentiated, may be benign or malignant, and are more common in males [19].

Benign teratomas are usually asymptomatic and many are discovered incidentally. Symptoms are mainly related to the mass effect of the tumor; hence they appear later as the tumor grows in the retroperitoneum and they include flank or abdominal pain, urinary symptoms as in this patient, and gastrointestinal symptoms such as constipation [16]. Physical examination may reveal abdominal mass or distention [16].

With the increased use of imaging, retroperitoneal teratomas increased in incidence. Plain radiographs may visualize calcifications but lead to nonspecific findings. Ultrasonography may be superior to radiography in delineating the cystic component but has limited specificity. Computed tomography scans and MRI are more appropriate. MRI can visualize the degree of invasion of adjacent structures as well as tumor content and may indicate if a wider resection is required [20, 21].

Complete excision of the mass is curative and is the mainstay of treatment since histological assessment is important for confirmation of the diagnosis and should be attempted since primary retroperitoneal teratomas in adults rarely infiltrate adjacent tissues [16]. In our case, histologic examination revealed all the characteristic features of dermoid cysts.

## 4. Conclusion

Mature (benign) retroperitoneal dermoid cysts compressing the prostate are extremely rare. Imaging should be considered when evaluating young male patients presenting with lower urinary tract symptoms. Surgical excision is the mainstay and gold standard treatment that allows symptomatic relief of the patient as well as definitive histologic diagnosis.
